# The Influence of the Plant Growth Regulator Maleic Hydrazide on Egyptian Broomrape Early Developmental Stages and Its Control Efficacy in Tomato under Greenhouse and Field Conditions

**DOI:** 10.3389/fpls.2017.00691

**Published:** 2017-05-16

**Authors:** Ariel Venezian, Evgenia Dor, Guy Achdari, Dina Plakhine, Evgeny Smirnov, Joseph Hershenhorn

**Affiliations:** ^1^Efal Agri Ltd.Netanya, Israel; ^2^Department of Plant Phytopathology and Weed Research, Newe Ya’ar Research Center, Agricultural Research OrganizationRamat Yishai, Israel

**Keywords:** *Solanum lycopersicum* L., *Phelipanche aegyptiaca*, parasitic weeds, chemical control, *Orobanche*

## Abstract

Broomrapes (*Phelipanche* spp. and *Orobanche* spp.) are holoparasitic plants that cause tremendous losses of agricultural crops worldwide. Broomrape control is extremely difficult and only amino acid biosynthesis-inhibiting herbicides present an acceptable control level. It is expected that broomrape resistance to these herbicides is not long in coming. Our objective was to develop a broomrape control system in tomato (*Solanum lycopersicum* L.) based on the plant growth regulator maleic hydrazide (MH). Petri-dish and polyethylene-bag system experiments revealed that MH has a slight inhibitory effect on *Phelipanche aegyptiaca* seed germination but is a potent inhibitor of the first stages of parasitism, namely attachment and the tubercle stage. MH phytotoxicity toward tomato and its *P. aegyptiaca*-control efficacy were tested in greenhouse experiments. MH was applied at 25, 50, 75, 150, 300, and 600 g a.i. ha^-1^ to tomato foliage grown in *P. aegyptiaca*-infested soil at 200 growing degree days (GDD) and again at 400 GDD. The treatments had no influence on tomato foliage or root dry weight. The total number of *P. aegyptiaca* attachments counted on the roots of the treated plants was significantly lower at 75 g a.i. ha^-1^ and also at higher MH rates. *Phelipanche aegyptiaca* biomass was close to zero at rates of 150, 300, and 600 g a.i. ha^-1^ MH. Field experiments were conducted to optimize the rate, timing and number of MH applications. Two application sequences gave superior results, both with five split applications applied at 100, 200, 400, 700, and 1000 GDD: (a) constant rate of 400 g a.i. ha^-1^; (b) first two applications at 270 g a.i. ha^-1^ and the next three applications at 540 g a.i. ha^-1^. Based on the results of this study, MH was registered for use in Israel in 2013 with the specified protocol and today, it is widely used by most Israeli tomato growers.

## Introduction

Broomrapes (*Phelipanche* spp. and *Orobanche* spp.) are holoparasitic plants that parasitize many dicotyledonous crops, causing tremendous losses in crop yield and quality worldwide (Joel et al., 2007). The parasite produces tiny seeds (0.2–0.3 mm) that remain viable in the soil for many years and germinate in response to substances—mostly are strigolactones—secreted from plant roots (Xie et al., 2010). The germinating seed produces a radicle that grows and reaches the root surface and produces a haustorium (the attachment stage). Intrusive haustorium cells penetrate the host root via enzymatic activity and physical force and form connections between the vascular systems of the host and parasite. The parasite develops into a tubercle that grows underground on the host root surface, sucking all of its nutrient needs from the host. Toward the end of its life cycle, the parasite develops flowering shoots that emerge aboveground, producing hundreds of thousands of seeds per plant (Parker and Riches, 1993).

Several methods were suggested for broomrape management that may be classified to resistant varieties, cultural, physical, biological, and chemical control by herbicides (Dhanapal et al., 1996; Ali, 2007). Resistant varieties and chemical control are probably the most commonly used commercially. Effective chemical control of broomrape is extremely difficult to achieve because these plants are anatomically and physiologically connected to the host. In many cases, when a broomrape sensitive crop is grown for the first time, the farmer is unaware of the parasite’s presence or infestation level in the field until broomrape shoots appear aboveground, at which point it is too late because most of the damage has already been done. In addition, control of aboveground shoots is much more difficult than at early developmental stages (Eizenberg et al., 2013). For effective control with systemic herbicides, the host should be highly selective to the herbicide without reducing the latter’s phytotoxicity as it passes through the host conductive tissues toward the roots (Joel et al., 2007). Broomrapes are chlorophyll-lacking parasites. Therefore photosynthesis-inhibiting herbicides cannot be used because there is no target site. To date, only amino acid biosynthesis-inhibiting herbicides have been used successfully to control broomrapes at a commercial level. These include imidazolinones, sulfonylureas, and glyphosate (Eizenberg et al., 2013). Imidazolinones and sulfonylureas inhibits acetolactate synthase (ALS), also known as acetohydroxy acid synthase (EC 4.1.3.18), a key enzyme in the biosynthesis of the branched-chain amino acids leucine, isoleucine and valine – HRAC group B (Herbicide Resistance Action Committee [HRAC], 2010). Glyphosate inhibits the enzyme 5-enolpyruvylshikimate-3-phosphate synthase (EPSPS; EC 2.5.1.19), which is essential for the synthesis of the aromatic amino acids phenylalanine, tryptophan and tyrosine – HRAC group G (Schönbrunn et al., 2001; Duggleby et al., 2008; Herbicide Resistance Action Committee [HRAC], 2010).

*Phelipanche aegyptiaca* is a devastating parasite on tomato (*Solanum lycopersicum* L., synonyms *Lycopersicon esculentum* Mill.), that endangers the existence of the processing tomato industry in Israel, among other countries (Hershenhorn et al., 2009). Sulfosulfuron (SS) has been registered in Israel for *P. aegyptiaca* control in tomato. Its usage involves three split applications with precise timing, each application followed by upper irrigation (Hershenhorn et al., 2009). The herbicide is not active when applied on tomato foliage, probably due to detoxification by a P450-type oxidase enzyme (Buker et al., 2004). To be effective, the herbicide should be washed into the soil so that it can establish direct contact with the germinating *P. aegyptiaca* seeds (Eizenberg et al., 2004). This method of control has several drawbacks. Any deviation from the prescribed application timing may drastically reduce control efficacy (Hershenhorn et al., 2009). Upper irrigation systems are no longer in use for tomato growth as all fields are drip-irrigated, so the farmer must invest in expensive irrigation equipment such as moving pivots. In addition, SS has a long residual effect in the soil, jeopardizing proper crop rotation. The upper irrigation, which enhances disease development, the expensive equipment needed, the precise timing and the long residual effect of SS in the soil considerably limit the number of fields in which this method is implemented in Israel.

Maleic hydrazide (MH; 1,2-dihydro-3,6-pyridazinedione) has been extensively used as a commercial systemic plant growth regulator and as herbicide since its introduction in 1949 (Schoene and Hoffman, 1949). After application to foliage, MH is freely translocated in plants to meristematic tissues, with mobility in both phloem and xylem (Meyer et al., 1987). Its mode of action in plants is not clear, although several hypotheses have been proposed and investigated, such as inhibition of cell division by mitotic disruption. Others have suggested that MH acts as an anti-auxin, anti-gibberellin or regulator of auxin metabolism and other plant growth regulators (Hoffman and Parups, 1964). Some carcinogenic effects of MH in mice, rats and cultured human lymphocytes raised a concern of its risks to man (Swietlińska and Žuk, 1978; De Marco et al., 1995; Ribas et al., 1996). MH is registered in the United States to control sprouting of potatoes and onions, suckers in tobacco, and growth of weeds, grasses, and trees in and along lawns, turf, ornamental plants, non-bearing citrus, utility and highway rights-of-way, airports and industrial land (Environmental Protection Agency [EPA], 1994). MH is also registered in Europe (except Austria, Denmark, Finland), Canada, Brazil, Argentina, China, Dominican Republic, Israel, Malaysia, South Africa, and other countries as well for sprout suppression on onion, shallot, garlic, carrots, and sprout suppression and control of volunteers on potatoes (Boyd, 2006; Coresta, 2014; European Food Safety Authority [EFSA], 2016). Previous attempts to use MH for broomrape control were reported in watermelon for *P. aegyptiaca* (Prokudina, 1976) and *P. aegyptiaca* and *P. ramosa* in tobacco (Evtushenko et al., 1973a; Danko, 1980; Lolas, 1986, 1994; Zhelev, 1988; Covarelli, 2002; Brault-Hernandez and Mornet, 2007) with moderate to high control efficacy. MH was also considered for dodder (*Cuscuta* spp.) control on sugar beet (Evtushenko et al., 1973b).

The limited number of effective herbicides for broomrape control and their extremely narrow range of modes of action, make it hard to match an herbicide to a crop plant for successful broomrape control. Glyphosate and ALS inhibitors are among the most widely used herbicides in the world, resulting in the appearance of glyphosate- and ALS-herbicide-resistant weed populations (Heap, 2014). This phenomenon greatly increases the chances of developing sulfonylurea-, imidazolinone-, and glyphosate-resistant broomrape races, because they are plants with high level of genetic flexibility, especially in areas where the herbicides are heavily used (Hershenhorn et al., 2009). Therefore, there is an urgent need to find new agents that effectively control broomrape by combining new modes of action with ease of use. MH’s mode of action differs from ALS and EPSPS inhibition and it has no residual soil effect.

The objectives of this work were: to study the effect of MH on early developmental stages of *P. aegyptiaca* and its translocation within tomato roots; and to optimize its ability to control of this parasitic weed in tomato cultivation under greenhouse and field conditions.

## Materials and Methods

### Plant Material

Processing tomato (*Solanum lycopersicom*) cv. M82 seeds were obtained from Tarsis Agricultural Chemicals Ltd. (Petah Tikva, Israel). *P. aegyptiaca* seeds were collected in 2015 from Egyptian broomrape inflorescences parasitizing tomato grown in Kibbutz Mevo Hama, in the Golan Heights, northern Israel (32°73′67″N, 35°65′52″E). The inflorescences were left to dry at 23–35°C for 2 months. The seeds were separated with a 300-mesh size sieve (50 micron) and stored in the dark at 4°C until use.

### Petri-Dish Experiment

The experiments was conducted according to Hershenhorn et al. (1997). *P. aegyptiaca* seeds were surface-sterilized in 70% ethanol solution for 1.5 min, transferred to 1% hypochlorite solution containing two drops of Tween 20 per 200 mL of water for 12 min, and washed three times with sterile water. The test was conducted in 45-mm-diameter Petri dishes, into which 8-mm-diameter GF/A glass microfiber filter paper disks (GFFP, Whatman International, Kent, UK) were placed on top of two layers of filter paper. Approximately 50 *P. aegyptiaca* seeds were placed on each disk. The Petri dishes were kept at 25°C for 5 days for preconditioning after which excess water was removed from the disks by blotting on a paper towel and they were transferred to new Petri dishes. Then 250 μL of the active isomer of GR24 (a synthetic strigol analog kindly provided by Prof. Yoneyama, Weed Science Center, Utsunomiya University, Utsunomiya, Japan) at a concentration of 10^-6^ M was added to each Petri dish. After 2 or 14 days the experiments were initiated by adding 30 μL of MH solution at concentrations of 1.4, 2.1, 2.8, 5.6, 11.2, or 22.4 mM to each Petri dish. Petri dishes receiving 30 μL water served as controls. Germ tube length of the radicle emerged from the germinating seeds was measured under a stereoscopic microscope at ×30 magnification 7 days after application (DAA) of water or MH. The Petri dishes were kept during the experiments in a growth chamber at 25°C in the dark. Each Petri dish contained five disks with three Petri dishes per treatment.

### Polyethylene-Bag (PEB) Experiments

*Phelipanche aegyptiaca* seeds were surface-sterilized as described above and dispersed on a 14 cm × 20 cm glass microfiber filter paper (70 seeds cm^-2^), equal to 20 000 seeds per 15 cm × 25 cm PEB into which the filter paper was inserted. A window (∼12 cm × 20 cm) was cut on one side of the bag face. The cuts were taped over with masking tape. A 1-month-old tomato seedling was planted in the PEB through the upper opening, with the root system scattered over the filter paper containing the broomrape seeds (Dor et al., 2007). Half strength Hoagland solution was used to supplement the plant’s growth in the PEB (Hoagland and Arnon, 1950). The bags were placed vertically in a paper folder covered on all sides with an opaque black polyethylene box to prevent light penetration into the root zone. The boxes were kept in a growth chamber at 25°C with 16 h/8 h day/night conditions, and a light intensity of 77 μEi m^-2^ s^-1^. Five milliliter of 10^-5^ M GR24 were applied 10 days after planting (DAP). Broomrape attachments appeared on the tomato roots 1 week after application (WAA) of GR. GR24 was used to get uniform and synchronized seed germination. At this stage, the herbicides 0.5 μM imazapic (IM), 10 μM SS, and 75, 150, 300, and 600 μM MH were injected onto the filter paper inside the PEBs. In a second PEB experiment, the same previous doses of IM, SS, and MH were applied at the tubercle stage, 2 WAA of GR24. Observations were carried out 7 and 14 DAA. The number of live and necrotic attachments on the surface of the filter paper in the PEBs was counted and the number of tubercles smaller and bigger than 3 mm was recorded under a stereoscopic microscope at ×30 magnification. Each treatment included five replicates (PEBs), each containing one tomato plant.

In the “double”-PEB system experiment, the plant’s roots were divided between two bags designated A – bag containing roots to which herbicide was applied, and B – bag containing the non-treated portion of the plant’s roots. The number of healthy and necrotic attachments was counted 14 DAA in bag A and B. Each treatment included five replicates (five double PEBs), each containing one tomato plant. Five milliliter of 10^-5^ M GR24 were applied 10 DAP. The herbicides IM (0.5 μM), SS (10 μM), and MH (600 μM) were injected onto the filter paper inside the bag A.

### Greenhouse Experiment

Tomato seeds were germinated in styrofoam growing trays and 4 weeks after sowing, were transferred to 2.0-L pots (one plant per pot) using soil from a field at Newe Ya’ar Research Center, Israel (medium-heavy clay–loam soil containing, on a dry wt. basis, 55% clay, 23% silt, 20% sand, 2% organic matter, pH 7.1), closely resemble soil characteristics of the field experimental plots except for a somewhat higher send content. Osmocote fertilizer (Osmocote^®^, Scotts Miracle-Gro, Marysville, OH, USA) at a concentration of 0.6% (w/v), and *P. aegyptiaca* seeds at a concentration of 15 mg seed kg^-1^ soil, (∼2250 seeds kg^-1^) were added to the soil. These components were mixed to homogeneity in a cement mixer for 10 min. Control pots did not contain *P. aegyptiaca* seeds. Soil temperatures in each experiment were recorded hourly throughout the experiment with temperature data logger (HOBO data logger, Onset Computer Corporation, Bourne, MA, USA) buried at one of the pots at 5 cm depth. The pots were placed in a greenhouse and drip-irrigated as needed. The experiments were arranged in a completely randomized design with four replications (pots) per treatment. MH at rates of 25, 50, 75, 150, 300, and 600 g a.i. ha^-1^ was applied twice at 200 (15 DAP) and 400 (30 DAP) growing degree days (GDD) on tomato foliage at 200 L ha^-1^ using a motorized sprayer equipped with a Tee Jet 8001E nozzle operated at a pressure of 300 kPa. Temperatures were converted to GDD as follows: GDD = Σ (*T_mean_* - *T_base_*), where *T_mean_* is the mean daily soil temperature (°C) and *T_base_* is the base temperature (°C). In this study, we used Dong et al. (2008) *T_base_* value for tomato of 10°C.

The experiment was terminated when broomrape inflorescences in the non-treated control pots started to develop seeds, about 7 WAA of the herbicide. The roots were gently washed out of the pot and the number of broomrapes, and fresh broomrape and root biomass were determined. Tomato plants were harvested and foliage fresh and dry weights (dried for 72 h at 72°C) were determined at the end of the experiment, 80 DAP.

### Field Experiments

Details of field experiments conducted between 2010 and 2012 at five locations in Israel (Ein Harod Ihud – three experiments, and one experiment each in Hulata, Mevo Hama, Kibbutz Mesilot and Havat Eden) are given in **Table 1**. All sites were plowed, cultivated and leveled, with 1.93-m-wide raised beds. Tomato plants were mechanically transplanted in two rows, 40 cm apart with 35 cm between plants in each row, and watered with the “Ra’am-Netafim” drip-irrigation system (Netafim Irrigation Equipment & Drip Systems, Kibbutz Hatzerim, Israel), placed between the two rows and comprised of a 17-mm diameter tube with drippers every 30–50 cm, each emitting 3.5 L h^-1^. Soil temperatures in each experiment were recorded hourly throughout the growing season with temperature data loggers buried at 5-cm depth in the middle of the experimental plot. The experimental plots were 2 m wide and 10 m long, replicated four times per treatment in a randomized block design. Pesticide and herbicide treatments for non-parasitic weeds were applied following the recommendations of the Israeli Extension Service. MH was applied using a backpack sprayer equipped with a 2-m spraying boom with Tee Jet 11015 nozzles (Spraying Systems Co., Wheaton, IL, USA) calibrated to deliver 200 L ha^-1^ at 300 kPa (Echo SHR210, Echo Ltd., Lake Zurich, IL, USA). MH application through the drip-irrigation system was conducted with a Fertilizing pump connected to the drip-irrigation pipes delivering 10 L h^-1^ and was conducted during the last hour of the irrigation period. Non-treated plots served as controls. MH application rates are given on **Tables 2**–**4**. In all experiments, tomato plants were harvested manually from a 5-m length of bed in the middle of each plot between late July and late September according to fruit and field conditions. *Phelipanche aegyptiaca* shoots were counted several times during the growing season.

**Table 1:** Details of field experiments conducted between 2010 and 2012 in five locations in Israel.

Location	Ein Harod Ihud			Hulata	Mevo Hama	Kibbutz Mesilot	Havat Eden

				Northern Israel			
				
Region	Jezreel Valley			Hula Valley	Golan Heights	Beit She’an Valley	

Coordinates	32°56′0.31″N,			33°05′0.29″N,	32°73′67″N,	32°49′74″N,	32°28′28″N,
	35°39′0.17″E			35°60′0.92″E	35°65′52″E	35°47′47″E	35°29′28″E
					
Year	2010	2011	2012	2010	2011	2012	2012
Soil	Medium-heavy			Medium clay	Medium-heavy	Medium-heavy	Medium-heavy
characteristics	clay loam soil			loam soil	clay loam soil	clay loam soil	clay loam soil
Clay (%)	57.0			46.1	60.5	48.3	51.0
Silt (%)	20.7			30.2	24.8	26.9	24.0
Sand (%)	13.1			11.7	6.7	12.4	9.0
CaCO_3_ (%)	8.9			9.1	7.1	10.5	14.8
Organic matter (%)	1.0			2.9	0.9	1.9	1.2
pH	7.1			7.3	7.1	7.4	7.1
Variety	5811	5811	LRT	8892	9205	5811	2549
			3715				
Planting date (dd.mm.)	10.03	15.03	28.02	11.05	02.05	23.02	22.02
Harvest date (dd.mm.)	05.07	04.07	05.07	–	10.08	18.06	14.06


**Table 2:** *Phelipanche aegyptiaca* control and tomato yield as influenced by five split applications of maleic hydrazide (MH) at various rates at Ein Harod Ihud and Hulata, 2010.

Treatment MH g a.i. ha^-1^ × no. of applications	*P. aegyptiaca* shoots per m^2^				Yield kg m^-2^
		
	Ein Harod Ihud		Hulata		Ein Harod Ihud
		
	Days after planting				
		
	75	85	63	76	
Control	12.4a	31.2a	3.8a	5.2a	6.0a
67.5 × 5	6.4ab	13.7a	2.7ab	2.7ab	6.7a
135 × 5	6.0ab	11.8ab	3.9a	5.2a	6.4a
270 × 5	3.8b	4.6b	0.7b	0.7b	7.6b
540 × 5	0.0c	0.0c	0.2b	0.2b	7.8b
67.5, 125, 192.5, 270, 540	6.4ab	6.6ab	0.5b	1.6ab	7.1ab


*In both experiments, MH was applied five times, 19 [197 growing degree days (GDD)], 32 (381 GDD), 46 (620 GDD), 60 (770 GDD), and 73 (980 GDD) days after planting (DAP) in Ein Harod Ihud and 21 (185 GDD), 35 (390 GDD), 49 (602 GDD), 64 (789 GDD), and 77 (990 GDD) DAP in Hulata. The data for each experiment were compared separately using Tukey–Kramer honest significant difference (HSD) test (*P* < 0.05) with JAMP program. Different letters indicate significant differences between treatments.*

**Table 3:** *Phelipanche aegyptiaca* control and tomato yield as influenced by one, two, three, or four split applications of MH at a constant rate of 540 g a.i. ha^-1^ at Ein Harod Ihud in 2011 and 2012 and at Mevo Hama in 2011.

Treatment	*P. aegyptiaca* shoots per m^2^			
	
MH g a.i. ha^-1^ × no. of applications	Ein Harod Ihud	Mevo Hama	Yield kg m^-2^	
	
	Days after planting		Ein Harod Ihud	Mevo Hama
		
	105	84	2011 and 2012	2011
			
	2011 and 2012	2011		
Control	28.9a	18.3a	7.4a	4.6a
540	22.5b	12.1b	8.6b	4.6a
540 × 2	11.7b	6.5c	9.0b	3.6a
540 × 3	1.1c	0.1d	9.3b	4.5a
540 × 4	1.0c	0.0d	9.1b	4.7a


*Maleic hydrazide was applied at 51 (355 GDD), 70 (548 GDD), 86 (740 GDD), and 103 (1000 GDD) DAP in Ein Harod Ihud and at 29 (400 GDD), 36 (627 GDD), 49 (817 GDD), and 77 (1020 GDD) DAP in Mevo Hama. The two experiments in Ein Harod Ihud (2011, 2012) were compared by Fisher’s *t*-test which showed homogeneity of variances, and therefore data were combined. The data for each experiment were compared separately using Tukey–Kramer HSD test (*P* < 0.05) with JAMP program. Different letters indicate significant differences between treatments.*

**Table 4:** *Phelipanche aegyptiaca* control and tomato yield as influenced by five split foliar applications of MH or three split applications through the drip-irrigation system at Kibbutz Mesilot and Havat Eden, 2012.

Time of application^a^								
	
100	200	400	700	1000	*P. aegyptiaca* shoots per m^2^		Yield kg m^-2^	
		
Days after planting					Days after planting		Mesilot	Havat Eden
		
24	38	53	73	90	Mesilot	Havat Eden		
		
Treatment					106	109		
MH g a.i. ha^-1^								
Control	25.0a	6.2a	2.8a	10.0a
270	270	540	540	540	0.1c	0.0b	8.7b	11.3b
400	400	400	400	400	0.9c	0.1b	7.1b	11.3b
	2700	2700	2700*^b^*		11.4b	2.3ab	5.4ab	8.2c
	2700	5200	7900*^b^*		9.0b	2.4ab	5.6ab	8.4c


*Data for each experiment were compared separately using Tukey–Kramer HSD test (*P* < 0.05) with JAMP program. Different letters indicate significant differences between treatments. ^a^Growing degree days. ^b^Applied through the drip-irrigation system.*

### Statistical Analysis

The Petri dish, PEB, greenhouse and field experiments were conducted twice except the experiment for the tomato yield as influenced by five split applications of MH at various rates which was conducted in three locations. The results were subjected to ANOVA using JMP software version 5.0 (SAS Institute Inc., Cary, NC, USA). To meet the assumption on ANOVA, percentage data were arcsine-transformed before analysis. On the graphs, back-transformed means are presented. The results were compared using Tukey–Kramer Honest Significant Difference (HSD) test (*P* < 0.05). The two experiments conducted at Ein Harod Ihud in 2011 and 2012 were compared using Fisher’s *t*-test, which showed homogeneity of variances, and therefore data were combined.

## Results

### Petri-Dish Experiments

**FIGURE 1:**
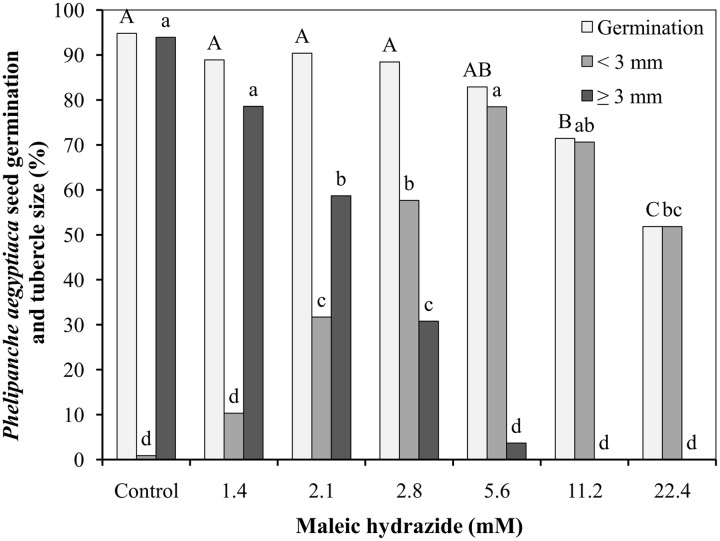
**Effect of maleic hydrazide (MH) on *Phelipanche aegyptiaca* seed germination in Petri dishes.** Experiments were initiated 2 days after the addition of 10^-6^ M GR24 by adding 30 μL water (control plates) or 30 μL MH solution at 1.4, 2.1, 2.8, 5.6, 11.2, or 22.4 mM (treated plates). Radical length of the germinating seeds was measured 7 days after water or MH applications. The results were compared using Tukey–Kramer honest significant difference (HSD) test (*P* < 0.05) with JAMP program. Uppercase letters indicate significant differences in *P. aegyptiaca* seed germination between treatments. Lowercase letters indicate significant differences in the number of tubercles shorter than 3 mm between treatments or in the number of tubercles longer than 3 mm between treatments. To meet the assumption on ANOVA, percentage data were arcsine-transformed before analysis. On the graphs, back-transformed means are presented.

The germination rate of *P. aegyptiaca* seeds after preconditioning and GR24 application was 95%, with over 95% of the germinated seeds producing germ tube longer than 3 mm 9 days after GR24 (or 7 days after MH) application to the Petri dishes. Germination rate was not influenced by the lower MH application rates of 1.4, 2.1, 2.8, and 5.6 mM, but it was significantly reduced at 11.2 mM, and even more drastically at 22.4 mM. At 1.4 mM, MH had no influence on seed germ tube length, but higher concentrations reduced their length in a concentration-dependent manner up to 5.6 mM. Almost all of the germinated seeds found in the Petri dishes treated with MH at 5.6 and all the germ tube of the germinated seeds at 11.2 and 22.4 mM were shorter than 3 mm (**Figure 1**).

### PEB System Experiments

**FIGURE 2:**
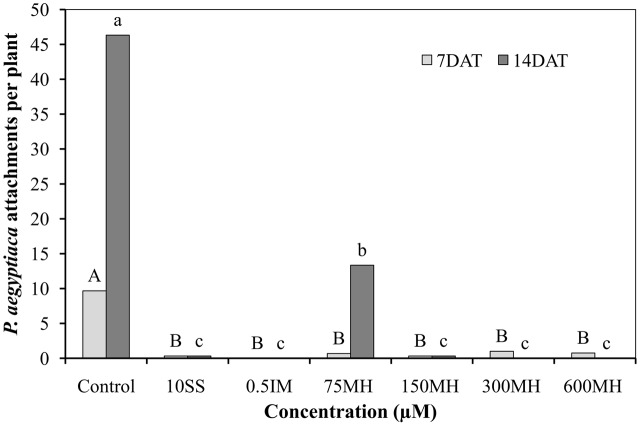
**Effect of MH and the herbicides sulfosulfuron (SS) and imazapic (IM) on *P. aegyptiaca* in the polyethylene-bag (PEB) system.** MH (75, 150, 300, and 600 μM), SS (10 μM), and IM (0.5 μM) were applied 2 weeks after the addition of 10^-5^ M GR24. The number of *P. aegyptiaca* attachments was counted 7 and 14 days after herbicide application (DAT). Data from each observation date were compared separately using Tukey–Kramer HSD test (*P* < 0.05) with JAMP program. Different letters indicate significant differences between treatments. Uppercase letters represent observations made after 7 days and lowercase letters, observations made after 14 days.

Imazapic (0.5 μM), SS (10 μM), and MH (75, 150, 300, and 600 μM) applied to PEBs 2 DAA of GR24 almost completely prevented the formation of *P. aegyptiaca* attachments on the roots when counted 7 days later. This situation did not change 14 DAA of the herbicides except for 75 μM MH, where 12 new attachments were found on the roots as compared to 46 in the control PEBs. Although 75 μM MH was still significantly reducing the number of new attachments 14 DAA (by 75% as compared to the control PEBs), it was significantly less effective than the higher concentrations of MH (150, 300, and 600 μM) and IM and SS at 0.5 and 10 μM, respectively, which kept the number of new attachments close to zero (**Figure 2**).

**FIGURE 3:**
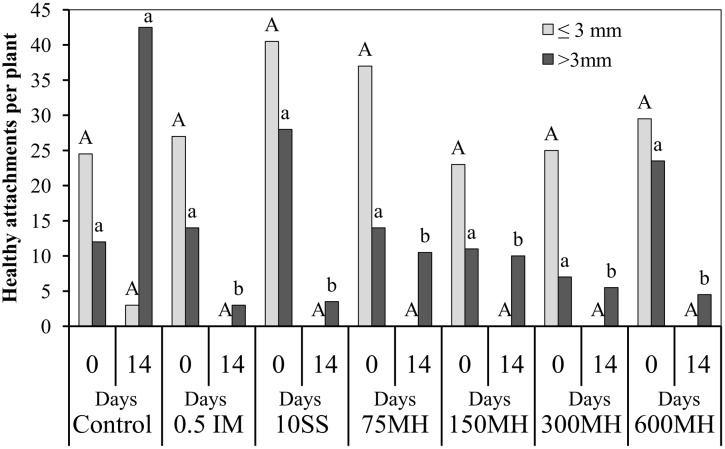
**Effect of MH and the herbicides SS and IM on *P. aegyptiaca* in the PEB system.** MH (75, 150, 300, and 600 μM), SS (10 μM), and IM (0.5 μM) were applied 2 weeks after the addition 10^-5^ M GR24. The number of *P. aegyptiaca* attachments was counted at the time of application (0) and 14 days later. Data for each observation date were compared separately by LSD on the basis of the Tukey-Kramer Honestly Significant Difference test (*P* < 0.05). Different letters indicate significant differences between treatments. Uppercase letters indicate significant differences in the number of tubercles shorter than 3 mm between treatments for the observation at time 0, and between treatments for the observation at 14 days. Lowercase letters indicate significant differences in the number of tubercles longer than 3 mm between treatments for the observation at time 0, and between treatments for the observation at 14 days.

In the second set of PEB experiments, MH, SS and IM were applied at the tubercle stage, 2 WAA of GR24. There was no difference in the number of tubercles at the time of application irrespective of their size. However, 14 DAA of herbicides, although there were still no differences in the number of tubercles smaller than 3 mm (0 in the herbicide-treated plants and only 3 in the control plants), there was a significantly lower number of tubercles bigger than 3 mm in the treated PEBs as compared to the controls (**Figure 3**).

**FIGURE 4:**
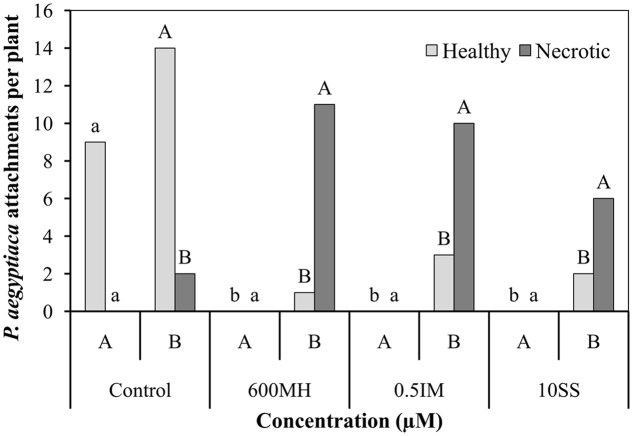
**Effect of MH, SS and IM on mortality of *P. aegyptiaca* attachments on tomato roots 2 weeks after their application in the double-polyethylene-bag system.** Each bag contained half of the tomato root system (Bag A – herbicide-treated PEB; bag B – non-treated PEB). Five ml of 10^-5^ M GR24 were applied to both bags 10 days after planting. MH, SS, and IM were applied only to bag A 1 week after the addition of GR24. Data from MH-treated and non-treated PEBs were compared separately using Tukey–Kramer HSD test (*P* < 0.05) with JAMP program. Different letters indicate significant differences between treatments. Uppercase letters refer to comparison of MH-treated PEBs, lowercase letters to the non-treated PEBs.

In the double-PEB system experiments, 2 WAA of herbicides, the following counts were obtained. In the non-treated control bags, there were 9 healthy tubercles and no necrotic ones in bag A and about 14 and 2 healthy and necrotic tubercles, respectively (90% healthy tubercles) in bag B. No healthy tubercles could be found in the herbicide-treated A bags and a high proportion of necrotic tubercles were found in the non-treated B bags, ranging from 92, 76, and 73% in the MH, SS, and IM bags, respectively (**Figure 4**).

### Greenhouse Experiments

**FIGURE 5:**
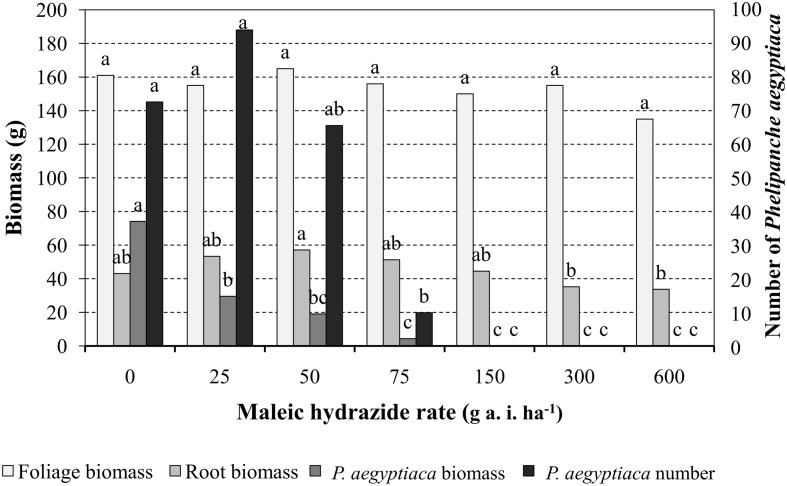
**The effect of MH applied to tomato foliage at 200 growing degree days (GDD) and 400 GDD on tomato foliage and root biomass and *P. aegyptiaca* number and biomass in pot experiments.** The experiments were terminated when broomrape inflorescences in the non-treated control pots began to develop seeds, about 7 weeks after the last herbicide application. Data for each parameter (tomato foliage and root biomass and *P. aegyptiaca* number and biomass) were compared separately using Tukey–Kramer HSD test (*P* < 0.05) with JAMP program. Different letters indicate significant differences between treatments.

Preliminary experiments were conducted in pots to determine MH phytotoxicity when applied on tomato foliage or directly into the pot soil. Results indicated that 1, 2, 3, or 4 split MH applications on the foliage or in the soil starting at 200 GDD with 200 GDD intervals at rates of 300, 450, or 600 g a.i. ha^-1^ had no effect on the plant’s visual appearance, height or weight or on fruit number or weight. MH applied at the indicated rates and timing directly to *P. aegyptiaca*-infested soil had no effect on the number of parasitic shoots or the time of their aboveground emergence. Foliar MH application experiments were repeated with tomato grown in *P. aegyptiaca*-infested soil treated at 200 GDD and again at 400 GDD with 25, 50, 75, 150, 300, or 600 g a.i ha^-1^ MH. The treatments had no influence on tomato foliage or root dry weight as compared to the non-treated plants. The total number of *P. aegyptiaca* counted on the roots of the treated plants was significantly lower at 75 g a.i. ha^-1^ MH and higher. Nevertheless, *P. aegyptiaca* biomass was significantly reduced at the lowest MH rate of 25 g a.i. ha^-1^ and further reduced, to close to 0, at the higher rates of 150, 300, and 600 g a.i. ha^-1^ MH (**Figure 5**).

### Field Experiments

#### Ein Harod Ihud and Hulata, 2010

The number of *P. aegyptiaca* shoots counted in the Ein Harod Ihud and Hulata experiments was significantly lower than in the control plots at the two highest MH rates (270 and 540 g a.i. ha^-1^) at the 75 and 63, and 85 and 76 DAP in Ein Harod Ihud and Hulata, respectively (**Table 2**). The tomato yield at Ein Harod Ihud was significantly higher than that in the control plots. No yield was harvested in Hulata due to pest attack on the last 2 weeks of the growth season. The number of broomrape was significantly lower at the plots treated with five MH applications of 270 or 540 or increasing application rates of 67.5, 125, 192.5, 270, and 540 g a.i. ha^-1^ at 63 DAP at Hulata but at the next observation, about 2 weeks later, only 5 applications of 270 or 540 g a.i. ha^-1^ MH caused a significant reduction in the number of *P. aegyptiaca* shoots between this treatment and the control.

#### Ein Harod Ihud 2011, 2012 and Kibbutz Mevo Hama 2011

At 540 g a.i. ha^-1^, MH was enough to significantly reduce the number of *P. aegyptiaca* shoots per m^2^ at both locations. Two applications at the same rate did not improve the control at Ein Harod Ihud but it did at Mevo Hama. Three and four split applications of the same rate further improved the control efficacy, with almost no *P. aegyptiaca* shoots at Mevo Hama and only 1 shoot m^-2^ at Ein Harod Ihud. The harvested yield in the Ein Harod Ihud experiments was significantly higher for all treatment applications, whereas at Mevo Hama, there was no detectable change in yield between the control and treated plots for any treatment (**Table 3**).

#### Kibbutz Mesilot and Havat Eden, 2012

Maleic hydrazide applied 5 times × 400 g a.i. ha^-1^ or in five split applications of 2 × 270 + 3 × 540 g a.i. ha^-1^ significantly reduced the number of *P. aegyptiaca* shoots to close to zero in the experiments conducted in Kibbutz Mesilot and Havat Eden in 2012. As a result, the yield was increased in both treatments and at both locations by an average of 1.3–5.9 kg m^-2^. Three split MH applications through the drip-irrigation system of 2700 g a.i. ha^-1^ (total of 8100 g a.i. ha^-1^) or three split applications at increasing rates of 2700, 5200, and 7900 g a.i. ha^-1^ (total of 15 800 g a.i. ha^-1^) reduced the number of *P. aegyptiaca* shoots significantly in Kibbutz Mesilot as compared to the control, but was less effective than five foliar split applications of 400 g a.i. ha^-1^ or five foliar split applications of 270 × 2 + 3 × 540 g a.i. ha^-1^. The treatments through the drip-irrigation system at Havat Eden were not effective and did not reduce *P. aegyptiaca* infection. The treatments through the drip-irrigation system had no effect on the yield at Kibbutz Mesilot and had a negative effect on the yield at Havat Eden (**Table 4**).

## Discussion

Maleic hydrazide has never been used for broomrape control in tomato, and its ability to control the parasite has thus never been compared to other relevant herbicides such as SS and IM (Eizenberg et al., 2013). The Petri-dish experiments were used to investigate MH’s effect on *P. aegyptiaca* seeds at early developmental stages, i.e., seed germination and germ tube development and elongation, before reaching the host root. MH demonstrated limited capacity to prevent seed germination. Only the two highest MH concentrations, 11.2 and 22.4 mM, were able to reduce the seed-germination percentage by 25 and 45%, respectively, as compared to the untreated controls. Lattice seed germination was reduced by 93% in response to 15 mM MH, (Haber and White, 1960), and Grant and Harney (1960) did not find any inhibitory effect on tomato seed germination at 10 mM MH, while *Albizia lebbeck* L. seeds responded in a positive germination rate to increase of MH concentration (Tomar, 2008). These results demonstrate the variation in seed sensitivity of different plant species to MH. Using dormant lettuce and y-irradiated wheat seeds, Haber and White (1960) concluded that MH inhibits cell division but has no effect on cell expansion. Similar experiments were conducted by Hershenhorn et al. (1998) with sulfonylurea herbicides. Chlorsulfuron, bensulfuron, primisulfuron, and thifensulfuron under similar conditions reduced seed germination rate by 17, 18, 25, and 19% at a concentration of 10 μM, which is 1000–2000 times lower than the MH concentrations used in our experiments. The same concentration, 10 μM, of seven phytotoxins—fusarenon X, nivalenol, deoxynivalenol, T-2 toxin, HT-2 toxin, diacetoxyscirpenol, and neosolaniol produced by *Fusarium* species caused 100% inhibition of *Phelipanche ramosa* seed germination (Zonno and Vurro, 2002).

Maleic hydrazide was much more efficient at preventing germ tube elongation. The two highest MH concentrations reduced germ tube length of all treated germinating seeds to less than 3 mm. Hershenhorn et al. (1998) reported that the sulfonylurea herbicides chlorsulfuron, bensulfuron, nicosulfuron, triasulfuron and thifensulfuron reduced germ tube length by ∼50% at a concentration of 10 μM. The fact that MH and the sulfonylurea herbicides were less effective at preventing seed germination and much more efficient at inhibiting seed germ tube elongation is not surprising, because most herbicides are active at the post-seed-germination stages rather than during initial seed germination phase (Cathey, 1964; Parka and Soper, 1977).

A PEB system was used to study the ability of MH, as compared to SS and IM, to prevent or inhibit the initial parasitism stages of *P. aegyptiaca*, namely, penetration and establishment in the host root tissues. In the first set of experiments, MH, SS, and IM were applied 2 days after the roots were exposed to GR24. The number of tubercles that formed on the control roots 7 DAA was high whereas in the treated plants, almost no tubercles formed, with no differences between the treatments. These results indicated very high control efficacy when the herbicides were applied shortly after the *P. aegyptiaca* seeds were triggered to germinate. These and the Petri-dish results suggest that the main MH inhibition mechanism is located at the penetration and establishment stages, because MH’s effect on the germination process in the Petri-dish experiments was rather limited. The observation made 14 DAA revealed no change in the treatments’ efficacy; except for 75 μM MH which, although somewhat less effective, still reduced tubercle number by 72% as compared to the control plants. To investigate the effect of the herbicides on a later stage of *P. aegyptiaca* parasitism, namely after tubercle formation, they were applied 2 WAA of GR24, when tubercles were already formed and were present on the roots. There were no significant differences between the treatments and the control plants in the number of healthy tubercles or their size at the time of herbicide application. However, all treatments significantly reduced the number of healthy tubercles and their size 2 WAA of herbicides. All treatments were equally effective, even the lower MH rate of 75 μM. This set of experiments demonstrated that MH can control *P. aegyptiaca* at the tubercle stage and is as effective as SS and IM, albeit at higher rates.

Translocation and movement of MH in the root system could enhance control efficacy if the compound flows, without being detoxified, into the *P. aegyptiaca* attachments, which serve as a strong sink (Fernández-Aparicio et al., 2016). The MH would then accumulate in the attachments until a lethal dose is achieved. To investigate MH translocation and flow in the roots, we used the double-PEB system. All attachments in the treated PEB were dead 2 WAA of herbicides regardless of the herbicide used—MH, SS, or IM. As expected, these results were in complete agreement with those obtained when using a PEB containing the whole plant root system. In the adjacent non-treated PEBs containing the other portion of the plant’s root system, 93, 77, and 75% of the attachments were dead in the MH, IM, and SS treatments, respectively. These results suggest that MH moves through the root system without being degraded to non-toxic metabolites. Uptake from solution of MH by barley roots and its translocation to shoots is pH dependent. Uptake and translocation was higher with the decrease of the solution pH probably by passive diffusion of the undissociated form of the acid. Translocation to shoots was approximately proportional to the chemical concentrations in the roots (Briggs et al., 1987). Radioactive labeled MH applied to leaves of *Zebrina pendula*, *Tradescantia fluminensis* and barley seedlings moved relatively free in both phloem and xylem (Crafts and Yamaguchi, 1958). MH absorption from *Ricinus communis* petioles was rather slow but was readily phloem-translocated from the mature leaves allowing appreciable quantities to reach the roots and the nutrient solution (Rigitano et al., 1987). MH absorption by foliar application, translocation to roots and between roots without being detoxified suggests an increase chances for successful broomrape control under greenhouse and field conditions.

Greenhouse experiments in pots revealed that MH up to 600 g a.i. ha^-1^ applied twice, at 200 and 400 GDD, has no negative effect on tomato foliage or root biomass, demonstrating that MH is very safe for use on tomato plants. Further applications were not conducted and fruit number and weight were not determined as the pot volume became the main limiting factor for plant growth. The total number of *P. aegyptiaca* counted on the roots was significantly lower at application rates of 75 g a.i. ha^-1^ or more as compared to the non-treated plants, and *P. aegyptiaca* biomass was reduced significantly at 25 g a.i. ha^-1^, the lowest rate tested. Those results prompted us to test MH control of *P. aegyptiaca* under field conditions.

Over 30 field experiments were conducted during 2010–2014 to optimize the timing, number and rates for maximal *P. aegyptiaca* control and tomato safety, as well as to minimize the total amount of MH applied. MH is registered in Israel for the prevention of onion and potato sprouting at rates of 2.2 and 2.5 kg ha^-1^, respectively. Our goal was to limit the total rate used for *P. aegyptiaca* control to this range, in order to facilitate MH registration for its new purpose—*P. aegyptiaca* control in tomato.

The first set of field experiments was aimed at detecting the most effective rate under a constant number of applications. We used five split applications following the application scheme established for *P. aegyptiaca* control in tomato with SS (Hershenhorn et al., 1998, 2009; Eizenberg et al., 2004, 2013; Ephrath et al., 2012), which dictates three split applications of sulfonylurea herbicides at 200 GDD intervals starting at 200 GDD, followed by two applications of IM, for a total of five split applications. A significant reduction of *P. aegyptiaca* shoots in the 2010 experiments at Ein Harod Ihud and Hulata was achieved with 270 g a.i. ha^-1^ MH and a further reduction with 540 g a.i. ha^-1^, a total of 1.35 and 2.7 kg a.i. ha^-1^ of MH, respectively. Gradually increasing the rates along the applications had no influence on *P. aegyptiaca* shoot number in Ein Harod Ihud but significantly decreased their number in Hulata at the early observation of 65 DAP; this effect then diminished and disappeared by 76 DAP. A positive correlation was found between *P. aegyptiaca* control and yield in Ein Harod Ihud. The yield was significantly higher with the treatments that were also effective at reducing the number of *P. aegyptiaca* shoots. Based on the results of these experiments, we further examined the minimal number of applications needed with 540 g a.i. ha^-1^, the most successful rate in the 2010 experiments.

We conducted three experiments aimed at testing the efficacy of an increased number of applications under a constant MH rate of 540 g a.i. ha^-1^. The results (**Table 3**) indicated that even one or two MH applications significantly reduce the number of *P. aegyptiaca* shoots. This was true for the two Ein Harod Ihud experiments whose results were combined. At Mevo Hama, two applications were more efficient than one application, which also reduced the number of *P. aegyptiaca* shoots significantly as compared to the non-treated control plots. In all three experiments, three and four MH applications gave superior results, with no aboveground *P. aegyptiaca* shoots in Mevo Hama and one aboveground *P. aegyptiaca* shoot per m^2^ in the Ein Harod Ihud experiments. The higher control efficacy achieved in Mevo Hama may be explained by the lower *P. aegyptiaca* infestation level in that field, with 18 shoot m^-2^ in the non-treated plots as opposed to about 30 shoot m^-2^ in the Ein Harod Ihud control plots. Although the infestation level in Mevo Hama was lower and the *P. aegyptiaca* control efficacy higher than at Ein Harod Ihud, the yield—irrespective of the control efficacy—was lower. This was probably a result of pest and disease outbreaks in the field toward the end of the growing season. All treatments in Ein Harod Ihud improved the yield regardless of the number of applications.

Maleic hydrazide at a rate of 700 g a.i. ha^-1^ applied on tobacco once at 40 DAP reduced the number of *P. ramosa* plants by 60–80% depending on the tobacco cultivar. The same rate applied twice at 40 and 60 DAP reduced the number of *P. ramosa* plants by 80–90% and led to a considerable yield increase (Lolas, 1986). Lower rate of 4500 + 4100 g a.i. ha^-1^ applied at 40 and 60 or 50 and 70 DAP resulted only in a moderate control (Lolas, 1994). Those results are in agreement with the results obtained in the present study were increased MH rates improved the control efficacy. However, Lubanov (1973) reported complete broomrape control with one MH application of 450 g a.i. ha^-1^ on tobacco at the bud formation stage. This complete control may be explained by the fact that the field was previously disinfected with metam-sodium. Similar results were also reported by Jelev (1988) were MH was applied once to tobacco 40 DAP. MH at 2000 g a.i. ha^-1^ applied once on tobacco foliage 55 DAP prevented completely the appearance of *P. ramosa* plants above ground but the same rate applied 20 days later had no effect on the number of *P. ramosa* plants as compared to the control plots (Covarelli, 2002) emphasizing the importance of the application timing. The main parameter dictating broomrape parasitism process and as a consequence herbicide application is the accumulated heat expressed as GDD (Joel et al., 2007; Hershenhorn et al., 2009; Eizenberg et al., 2013). Comparison to our results cannot be conducted without available GDD data. However, it should be noted that MH early application had phytotoxic effect on tobacco plants. In our experiments one MH application of 540 g a.i. ha^-1^, only 25% of the rate used by Covarelli (2002), 20 DAP reduced the number of *P. aegyptiaca* plants by 12% in the two experiments conducted at 2011 and 2012 at Ein Harod Ihud and by 34% in Mevo Hama without any phytotoxic effects. The host plant roots grow and penetrate in to deeper soil layers during the growing season and stimulate new broomrape seeds to germinate. Therefore repeated MH applications are needed to effectively control the parasite during the whole growing season. It may be hypothesized that complete control of the parasite by one MH application may occur if MH stays at an effective lethal concentration level in the tobacco roots during all the growing season. These contrasting results demonstrate the problematic of comparing experiments conducted in different crops under different conditions. Differences in physiology and morphology of the host plant species, soil type, environmental conditions such as temperature and rainfall, irrigation regime and equipment, growth conditions and as a result plant status and stress, broomrape species and sampling methods, spraying equipment and MH formulation and surfactants, infestation level and cultivation practices are only some of the factors which influence control efficacy.

In irrigated water melon a reduction of the infestation level by 67–96% was achieved with MH at 5000 g a.i. ha^-1^ repeated twice at 10–20 days (Prokudina, 1976). Such high MH rates were not tested in our experiments. In our preliminary experiments one MH application of 1100 g a.i. ha^-1^ caused a significant damage to tomato plants (curly and silvery leaves, flower drops and as a result extensive vegetative growth) and therefore were not continued to be tested (Sarkar et al., 2014).

In the last set of experiments conducted at Kibbutz Mesilot and Havat Eden in 2012 (**Table 4**), we tried two different options to reduce the total MH amount applied to the field during the growing season. Since we had found, in preliminary pot experiments, that moving the first application to 100 GDD has no negative effect on the tomato plant, we reduced the rate of the two first applications to 270 g a.i. ha^-1^ and started the first application at 100 GDD. The second option was a constant rate of 400 g a.i. ha^-1^, also starting at 100 GDD. As MH may be absorbed by roots and was proven in our double-PEB experiments to flow through the root system to *P. aegyptiaca* attachments, we applied a very high amount of MH through the drip-irrigation system. Five foliar applications, regardless of rate, had excellent control efficacy, almost completely preventing the appearance of *P. aegyptiaca* shoots aboveground. This was especially impressive at Kibbutz Mesilot as the infestation level was high. MH applied three times through the drip-irrigation system had no effect on the number of *P. aegyptiaca* shoots in Havat Eden, and three split applications of 2700, 5200, and 7900 g a.i. ha^-1^ reduced shoot numbers significantly as compared to the non-treated plots but were not as effective as the foliar treatments. The effective control of the foliar applications resulted in a significant yield increase in both locations. The drip-irrigation applications had no effect on yield at Kibbutz Mesilot and a negative effect at Havat Eden. The inefficiency of MH control when applied through the soil, even at the very large amounts applied in our experiments, may be explained by the soil characteristics. Although MH is readily absorbed by roots, it rapidly disappear form soil (Levi and Crafts, 1952; Hoffman et al., 1962; Helweg, 1974). There are several soil components that enhance MH adsorption and inactivation, such as clay, specific surface area and pH, but not organic matter content (Hermosin et al., 1987) The end result is less herbicide available to the plant roots and therefore to *P. aegyptiaca*.

Based on these results and many other field and observation trials, MH was registered in March 2013 for *P. aegyptiaca* control in processing tomato fields in Israel under the two protocols presented in **Table 4**. Since its registration, MH has been widely used by Israeli tomato growers with excellent control results, crop safety and yield increases.

## Author Contributions

AV contributed to the conception of the work, conducted the experiments and analyzed the data, ED conducted the statistical analysis, contributed to data interpretation and edited the manuscript, GA conducted the experiments, DP conducted the experiments and contributed to the design of the work, ES conducted the experiments, JH planned the study, analyzed and interpreted the data, drafted the manuscript and ultimately approved the version to be published.

## Conflict of Interest Statement

The authors declare that the research was conducted in the absence of any commercial or financial relationships that could be construed as a potential conflict of interest. The reviewer BC and handling Editor declared their shared affiliation, and the handling Editor states that the process nevertheless met the standards of a fair and objective review.
